# Generation of Yellow Fever virus vaccine in skeletal muscle cells of chicken embryos

**DOI:** 10.1590/0074-02760190187

**Published:** 2019-12-09

**Authors:** Yuli Rodrigues Maia de Souza, Pedro Paulo de Abreu Manso, Barbara CEP Dias de Oliveira, Márcia Andreia Barge Loução Terra, Thalita Paschoal, Giulia Caminha, Ieda Pereira Ribeiro, Lidiane Menezes Souza Raphael, Myrna Cristina Bonaldo, Marcelo Pelajo-Machado

**Affiliations:** 1 Fundação Oswaldo Cruz-Fiocruz, Instituto Oswaldo Cruz, Laboratório de Patologia, Rio de Janeiro, RJ, Brasil; 2 Fundação Oswaldo Cruz-Fiocruz, Instituto Oswaldo Cruz, Laboratório de Biologia Molecular de Flavivirus, Rio de Janeiro, RJ, Brasil

**Keywords:** Yellow Fever, vaccine, 17DD, chicken embryos, muscle cell, cell culture

## Abstract

**BACKGROUND:**

The Yellow Fever (YF) vaccine is produced by the inoculation of embryonated chicken eggs with YF17DD virus on the ninth day of development. Full embryos are collected on the twelfth day of development for vaccine formulation. Skeletal muscle tissue is the main site where biosynthesis of viral particles occurs.

**OBJECTIVES:**

This study aimed to analyse the experimental infection of skeletal muscle cells of chicken embryos by the 17DD Yellow Fever virus (YFV) *in vivo* and *in vitro*.

**METHODS:**

Chicken embryos infected with YF17DD virus were analysed by immunofluorescence using confocal and super-resolution microscopes. Primary cultures of skeletal muscle cells of non-infected chicken embryos were evaluated for susceptibility and permissiveness to YF17DD virus using different protocols. This evaluation was performed based on morphological, viral titration, molecular biology, and colorimetric techniques.

**FINDINGS:**

The present work phenotypically characterises embryonic chicken skeletal muscle cells as myogenic precursors expressing the Pax7 transcription factor in some cases. We demonstrated that these cells are susceptible to *in vitro* infection at different multiplicities of infection (MOIs), reproducing the same infection pattern observed *in vivo*. Furthermore, myogenic precursors and myoblasts are preferred infection targets, but establishment of infection does not depend on the presence of these cells. The peak of viral production occurred at 48 hpi, with decay occurring 72 hpi, when the cytopathic effect can be observed.

**MAIN CONCLUSIONS:**

In conclusion, the primary culture of chicken skeletal muscle cells is a good model for studying muscle cells infected with YF17DD virus. This culture system displays satisfactory emulation of the *in vitro* phenomenon observed, contributing to our understanding of virus infection dynamics and leading to the development of alternative methods of vaccine production.

The Yellow Fever virus (YFV) is a member of the *Flaviviridae* family that includes dengue, West Nile, Japanese encephalitis, and Zika viruses. Many flaviviruses are pathogenic to humans and are transmitted by arthropods, particularly by mosquitoes.^1^ Yellow fever (YF) affects predominantly Sub-Saharan African and South American populations. Most affected individuals present mild symptoms similar to those observed in those with influenza and rarely develop the severe form of the disease. Patients with severe YF present fever, haemorrhage, shock, and injuries to the kidney, liver, and myocardial tissue; their mortality rate is approximately 50%.^2^ The best way to prevent YF is by mass vaccination of people who live in, or who will travel to, areas where the disease is endemic. YF vaccine is highly effective, offering lifetime protection with a single dose.^3^ Despite this, YF remains a major cause of morbidity and mortality in Africa and South America. Most of those affected are inhabitants of endemic and epizootic areas, unvaccinated travellers, or people to whom the vaccine is contraindicated, such as those with allergies to chicken egg proteins, children younger than 9 months old, adults more than 60 years old, and immunosuppressed individuals.^3^ ^,^ ^4^ ^,^ ^5^

YF vaccine is produced by the inoculation of substrain YF17DD virus into embryonated chicken eggs according to standard protocols established by the World Health Organization.^6^ Although chicken embryos have been used since 1937 as biological systems for the production of YFV,^2^ tissues and cell targets responsible for the biosynthesis of viral particles have only been described very recently.^7^ Moreover, skeletal muscle is the main tissue infected in chicken embryos, suggesting that muscle cells are a major site for production of viral particles after infection.^8^

In the present study, we analysed the musculoskeletal tissue of chicken embryos infected by YF17DD virus to better characterise the first infected cells. We also evaluated the *in vitro* YF17DD infection of primary embryonic chicken skeletal muscle cell cultures, defining the best conditions for the establishment of *in vitro* infection in the culture system, including kinetics of viral production as well as the peak of viral production. Both *in vivo* and *in vitro* processes of viral infection were similar in terms of early cell targets and evolution of tissue infection. Our data contribute to the understanding of infection in chicken embryos, supporting the development of an alternative method for producing the YF attenuated vaccine.

## MATERIALS AND METHODS

*Histopathological analysis* - Embryonated eggs were infected in the yolk sac with 17DD EPlow virus seed lot (1-5 x 10^3^ PFU per inoculum) on the ninth day of embryo development. Eggs were kept in an IP70 brooder (Premium Ecologica, Brazil) with controlled temperature at 37.5ºC and 55% relative air humidity. For negative controls, embryos kept under the same conditions were inoculated in their yolk sacs with water. All embryos were euthanised at 48 h post-infection (11 days of development). The experiment was carried out using three infected embryos and the same number of controls.

Embryo trunks were cleaved transversely in 3 mm sequential sections and separated from the head, wings, and legs. All samples were fixed in Carson-Millonig formalin^9^ for 48 h and processed according to standard histological techniques for paraffin embedding. At least three sections (5 μm thick) from each block were evaluated by immunofluorescence assay.

*Cell culture establishment and infection* - Primary cultures of myogenic cells were prepared from breast and leg muscles of non-infected 11-day-old chicken embryos. A pool of cells from 3-6 embryos was used to perform the cultures. Tissues were collected, dissected out, and incubated at 37ºC for 10 min in 0.05% trypsin solution (Gibco). After removal of trypsin, the resulting suspension was filtered, and cells were plated at a density of 4 × 10^5^ cells/1.9 mm culture dish onto coverslips previously coated with 2% gelatin. Muscle cells were maintained in minimal essential media (MEM) enriched with 10% horse serum, 5% chicken embryo extract, 100 U/mL penicillin, and 100 µg/mL streptomycin. After culture establishment, cells were exposed to YF17DD virus extracted from infected embryos provided by the vaccine production unit (Biomanguinhos). Four points of infection were performed: at seeding and at 5, 24, and 120 h after plating (designated T0, T5, T24, and T120, respectively). T0 cultures were exposed to YF17DD virus immediately after myogenic precursors and myoblasts were removed from tissues. T5 cultures were exposed to YF17DD virus when those myogenic precursors and myoblasts adhered to coverslips. In T24 cultures, young fibres were exposed to YF17DD virus when most muscle cells differentiated.^10^ T120 cultures were exposed to YF17DD virus when differentiated fibres and fibroblasts were detected in the culture, with less differentiated muscle cells.^10^ At each time point, medium was removed, and cells were infected with virus at 0.1, 0.01, and 0.002 multiplicity of infection (MOI) by adsorption at 37ºC for 1 h. Medium with virus was then removed and exchanged for fresh complete MEM.

*Immunofluorescence assay* - Sections of all paraffin blocks were evaluated by immunofluorescence assay, as previously described.^7^ Three coverslips each from infected and control cell cultures were fixed in Carson-Millonig formalin^9^ for 10 min at 25-30ºC and permeabilised with 0.1% Triton X-100 (Merck, USA). Slides and coverslips were incubated with a mouse polyclonal antibody (anti-YFV, Evandro Chagas Institute, Brazil). Double or triple staining was performed using anti-desmin (cat. RB-9014, Thermo Scientific, USA), anti-myosin (cat. Ab124205, Abcam, USA) and anti-Pax7 antibodies (Developmental Studies Hybridoma Bank, USA). Secondary antibodies AlexaFluor 488-conjugated goat anti-mouse (cat. A11001, Invitrogen, USA), AlexaFluor 546-conjugated goat anti-mouse (cat. A11003, Invitrogen), or AlexaFluor 635-conjugated goat anti-rabbit (cat. A31576, Invitrogen) was applied at 37ºC for 1 h, followed by counterstaining with 1:5000 DAPI (cat. 03571, Molecular Probes, USA). Negative controls were processed by omitting treatment with primary antibodies. All sections and coverslips were analysed under the fluorescence microscope Axio Observer Z1 coupled with a Colibri system (Carl Zeiss, Germany). Selected fields were analysed under a laser scanning microscopes (LSM) 710 confocal microscope (Carl Zeiss) and an Elyra PS.1 super-resolution microscope (Carl Zeiss).

*Viral titration* - Supernatants were taken from control and from infected cultures, three samples of each were combined to make pooled control and infected groups per experiment, and experiments were performed in triplicate. The control and the infected pooled samples were evaluated using the plaque-forming unit assay (PFU). Vero cells were plated at a density of 1 x 10^5^ cells/mL in 24-well plates, and supernatants were serially diluted from 10^-1^ to 10^-6^ in Earles’s 199 medium (Thermo Scientific). After 1 h incubation, the inoculum was removed and wells were overlaid with Earles’s 199 medium with 3.5% carboxymethylcellulose (Merck), 5% NaHCO_2_, and 5% foetal bovine serum (FBS) (Thermo Scientific). After seven days of incubation at 37ºC and 5% CO_2_, cells were fixed in 10% formaldehyde and subsequently stained with 0.01% crystal violet. Results obtained were expressed in PFU/mL. Statistical analysis was performed using the Kruskal-Wallis test and GraphPad software.

*Polymerase chain reaction (PCR)* - RNA samples were extracted from cell culture dishes, either positive or negative for YF17DD virus, with Trizol according to the manufacturer’s recommendation (Thermo Fisher, USA). Then 0.1 mL chloroform per sample was added, followed by 100% isopropanol, 70% ethanol and ultrapure water, with washes between steps. Samples eluted after the procedure were amplified by reverse transcription-polymerase chain reaction (RT-PCR) (Thermoscript RT-PCR kit, cat. 11146016, Life Technologies, USA) with universal flavivirus primers described by Tanaka^11^ (YF1-5ʹ-GGTCTCCTCTAACCTCTAG-3ʹ and YF3-5ʹ-GAGTGGATGACCACGGAAGACATGC-3ʹ). At the end, amplicons were subjected to electrophoresis. Samples with 675 bp bands were considered positive.

*Cell density assay* - Chicken embryo skeletal muscle cells infected in culture with YF17DD virus at 0.002 MOI and non-infected (controls) were evaluated for cell density at 24, 48, and 72 h post infection (hpi). After removing culture medium, cells were fixed for 10 min in 3.7% Carson buffered formalin^9^ at room temperature and washed twice in phosphate buffered saline (PBS). Cells were then stained with 200 μL of 1% crystal violet solution for 10 min, also at room temperature. Following staining, samples were washed with several exchanges of distilled water. Dye was extracted with 200 μL absolute methanol for 10 min. Afterwards, 100 μL of this supernatant was collected, placed in a 96-well plate, and analysed in a spectrophotometer at 595 nm.

*Ethics statement* - Specific pathogens-free (SPF) fertilised White Leghorn chicken eggs (*Gallus gallus domesticus*; Linnaeus, 1758) were obtained from the YF vaccine production unit (Instituto de Tecnologia em Imunobiológicos ― Biomanguinhos, Fiocruz, Rio de Janeiro, Brazil). All experiments were in accordance with the YF vaccine production protocol, applied since 1937 when vaccine production started at Biomanguinhos, under the ethical approval of Fiocruz. In addition, all procedures involving animal experimentation were performed as approved by the Ethics Committee (CEUA/IOC), under license numbers: L-025/2017 and L-028/2017.

## RESULTS

**Fig. 1:**
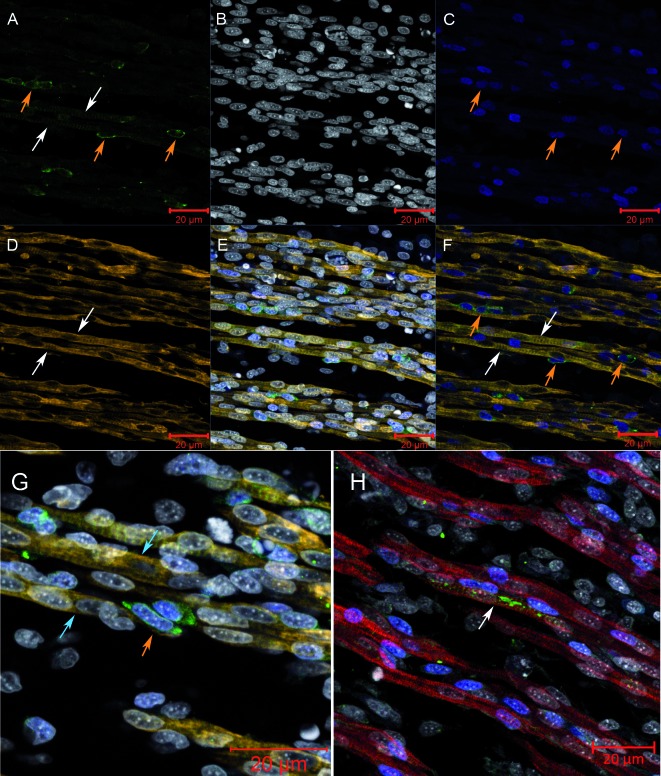
myogenic precursors infected with the YF17DD virus are observed close to infected and non-infected muscle fibres. Skeletal muscle tissue of chicken embryos infected with YF17DD virus at 9 dd and collected at 48 hpi were subjected to immunofluorescence assay and analysed using a confocal laser microscope. Pax-7^+^ mononuclear infected cells fusing to infected fibres were observed in infected embryos. Yellow Fever virus (YFV) antigens (green - A), nucleus (white - B), Pax7 (blue - C), desmin (yellow - D), merge YFV, nucleus, Pax7 and desmin (E) and merge YFV, Pax7 and desmin (F). Infected Pax7^+^ mononuclear cells were observed near non-infected muscle fibres. Desmin (yellow), YFV (green), Pax7 (blue), and nucleus (white) (G). Infected muscle fibres present virus staining accompanying fibre striation. Myosin (red), YFV (green), Pax7 (blue) and nucleus (white). Pax7 cells - orange arrows, infected fibres - white arrows, non-infected cells - blue arrows.

**Fig. 2:**
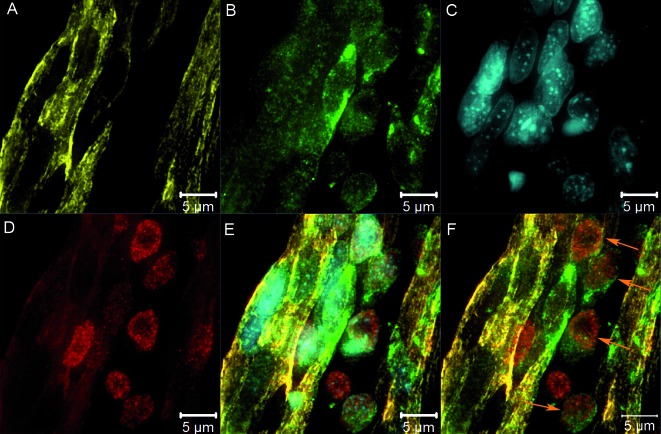
infected mononucleated cells are Pax7^+^. Skeletal muscle tissue from chicken embryos infected with YF17DD virus at 9 dd and collected at 48 hpi were subjected to immunofluorescence assay and analysed by structured illumination microscopy (SIM) super resolution microscopy. Detail of infected mononuclear cell fusing to the muscular fibre. Desmin (yellow -A), Yellow Fever virus (YFV) (green -B), nucleus (blue - C), Pax7 (red - D), merge YFV, nucleus, Pax7 and desmin (E) and merge YFV, Pax7 and desmin (F). Pax7 infected cells - orange arrows.

*Immunophenotyping of skeletal muscle cells at initial stage of infection* - Immunofluorescence assay of skeletal muscle at 48 hpi showed that the first cells to appear infected were Pax7^+^ (Fig. 1A-G, orange arrows). These cells were observed between infected (Fig. 1A, D, F, white arrows) and non-infected (Fig. 1G, blue arrow) muscle fibres, sometimes apparently fused with them (Fig. 2A-F). Viral particles accompanying the fibre striation pattern were also observed (Fig. 1H).

**Fig. 3:**
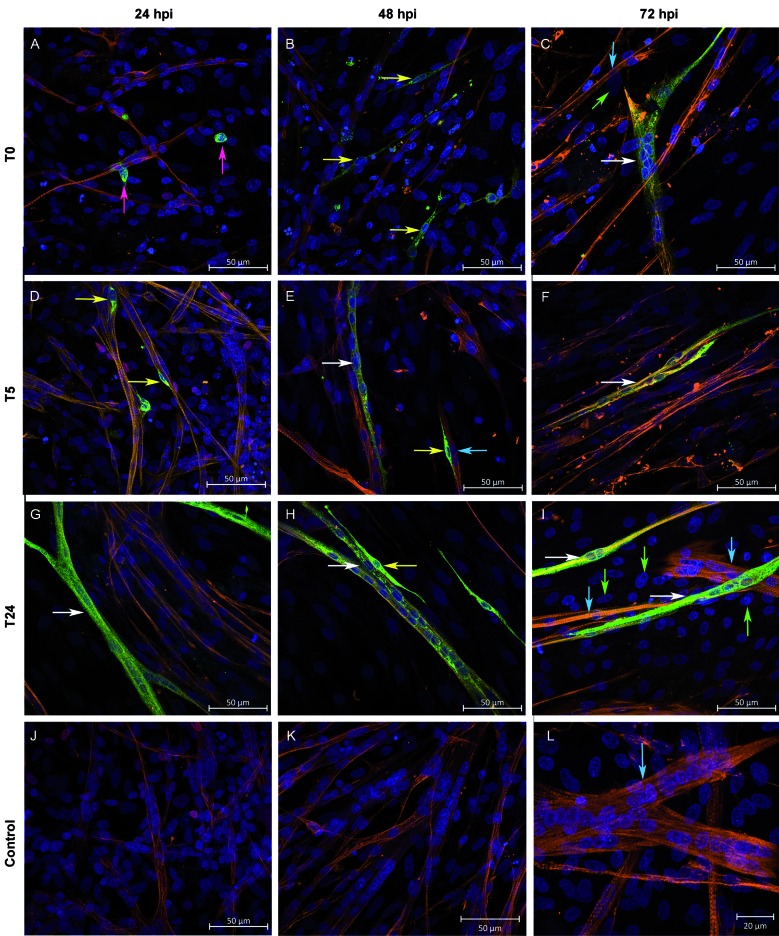
muscle chicken cells are susceptible to *in vitro* infection in different stages of differentiation. Skeletal muscle cells cultured and infected *in vitro* by the Yellow Fever virus (YFV) 17DD at 0.1 multiplicity of infection (MOI) were subjected to immunofluorescence assay and analysed using a confocal laser microscope. Cells infected at the moment of plating (T0) at 24 (A), 48 (B), and 72 hpi (C). Cells infected 5 h post-plating (T5) at 24 (D), 48 (E), and 72 hpi (F). Cells infected 24 h post-plating (24) at 24 (G), 48 (H), and 72 hpi (I). Control culture non-infected at 24 (J), 48 (K) and 72 hpi (L). Desmin (orange), YFV (green) and nucleus (blue). Myoblasts - pink arrows, Myocytes - yellow arrows, infected fibres - white arrows, non-infected fibres - blue arrows, fibroblasts - green arrows.

*In vitro exposure of skeletal muscle cells to YFV in different stages of differentiation* - Primary muscle chicken embryo cell cultures were susceptible to YF17DD virus in all infection protocols (T0, T5, T24, and T120). For these experiments, cells were infected with 0.1 MOI. Under all conditions, some infected myoblasts (Fig. 3A, pink arrow), myocytes (Fig. 3D, yellow arrow), and new fibres (Fig. 3G, white arrow) at 24 hpi were observed. We observed a marked increase in the number of myocytes (Fig. 3B yellow arrow) and infected fibres (Fig. 3E, white arrow), with infected myocytes trying to fuse to non-infected (Fig. 3E, yellow and blue arrows) and infected muscle fibres (Fig. 3H, yellow and white arrow), observed at 48 hpi. At 72 hpi, some infected muscle fibres (Fig. 3C, F, I, white arrows) thinner than controls (Fig. 3L, blue arrow), and uninfected fibroblasts (Fig. 3C, I, green arrows) accompanied by a few uninfected muscle fibres (Fig. 3C, I, blue arrows) were observed.

**Fig. 4:**
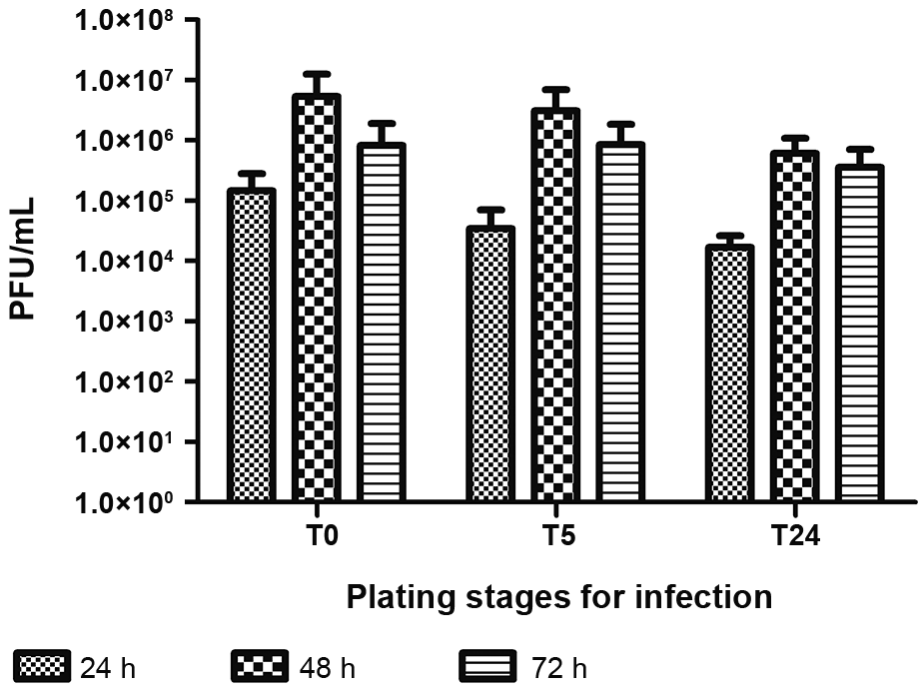
muscle cells in culture infected at different stages of differentiation produce infectious viral particles. Chicken embryo muscle cells infected at 0.1 multiplicity of infection (MOI) at plating stage (T0), after 5 h (T5), and after 24 h (T24) were evaluated 24, 48, and 72 hpi titrated using the plaque-forming unit assay (PFU) technique. Each bar represents a pool of three samples in triplicate.

Corroborating morphological data, initial production of infectious particles at 24 hpi was observed. The highest level of production was observed after 48 hpi, followed by a small decrease of viral particle production at 72 hpi. Infection at T0 presented the most efficient production compared to T5 and T24 (Fig. 4).

**Fig. 5:**
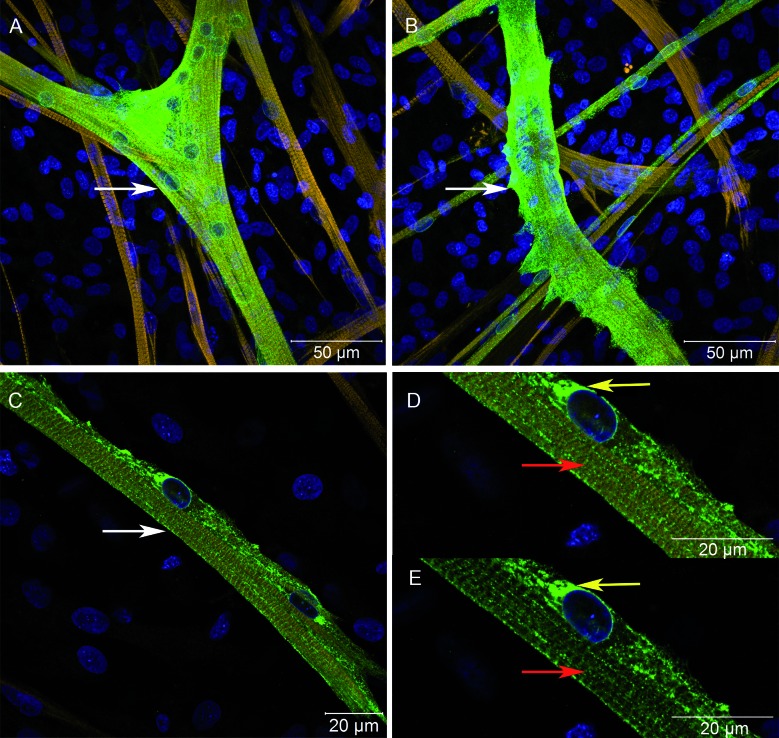
myofibers are susceptible to infection by the YF17DD virus, and viral proteins intercalate with myosin labelling. Skeletal muscle cells from chicken embryos cultured and infected *in vitro* with the Yellow Fever virus (YFV) 17DD at 0.1 multiplicity of infection (MOI) 168 h after plating ((T120)/48 hpi) were subjected to immunofluorescence assay and analysed using a confocal laser microscope. Huge myosin-positive fibres were strongly infected by YFV (A-C). Detail of viral proteins following fibre striations, with perinuclear deposition (D and E). Myosin (yellow), nucleus (blue) and YFV (green). Muscle fibres - white arrows, YFV following fibre striation - red arrows, YFV perinuclear deposition - yellow arrows.

*Role of myogenic precursors and myoblasts at in vitro infection establishment* - To determine if myogenic precursors and myoblasts were essential for establishing *in vitro* infection, T120 cultures were exposed to YF17DD virus at 0.1 MOI. In this protocol, muscle cell cultures were totally differentiated and mature, with robust and large fibres, and muscle cells were also susceptible to infection, even in the absence of myogenic precursors and myoblasts (Fig. 5A-C, white arrows). Viral proteins located along fibre striations (Fig. 5D-E, red arrows) and showing perinuclear deposition (Fig. 5D-E, yellow arrows) were observed at 48 hpi.

**Fig. 6:**
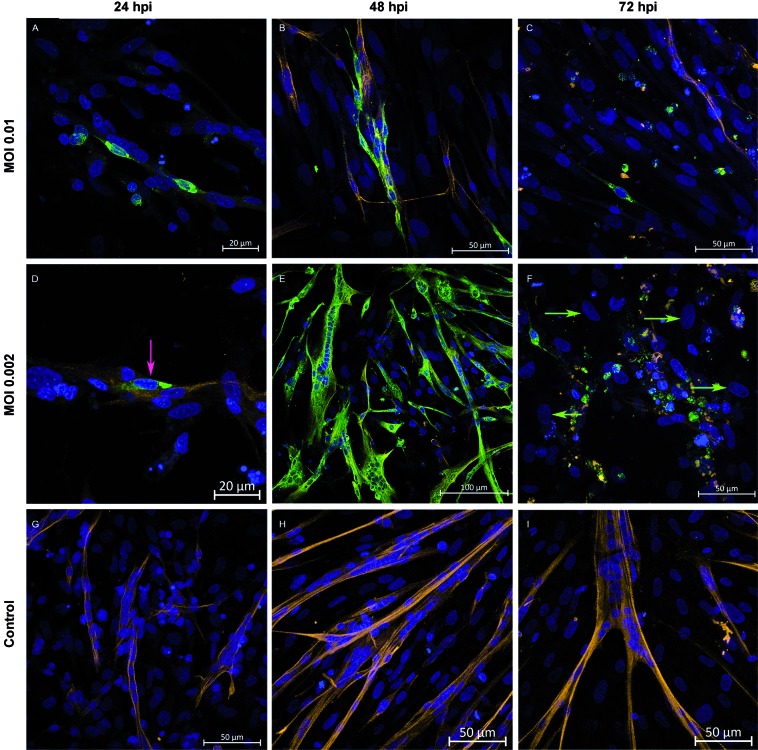
i*n vitro* infection of chicken muscle cells is more intense at 0.002 multiplicity of infection (MOI). Skeletal muscle cells cultured and infected *in vitro* at different MOIs by the yellow fever virus 17DD at the moment of seeding (T0) were subjected to immunofluorescence assay and analysed using a confocal laser microscope. Infection was performed with: 0.01 MOI at 24 (A), 48 (B), and 72 hpi (C); 0.002 MOI at 24 (D), 48 (E), and 72 hpi (F). Control culture non-infected at 24 (G), 48 (H), and 72 hpi (I). Desmin (orange), YFV (green) and nucleus (blue). Myoblast - pink arrow, fibroblasts - green arrows.

**Fig. 7:**
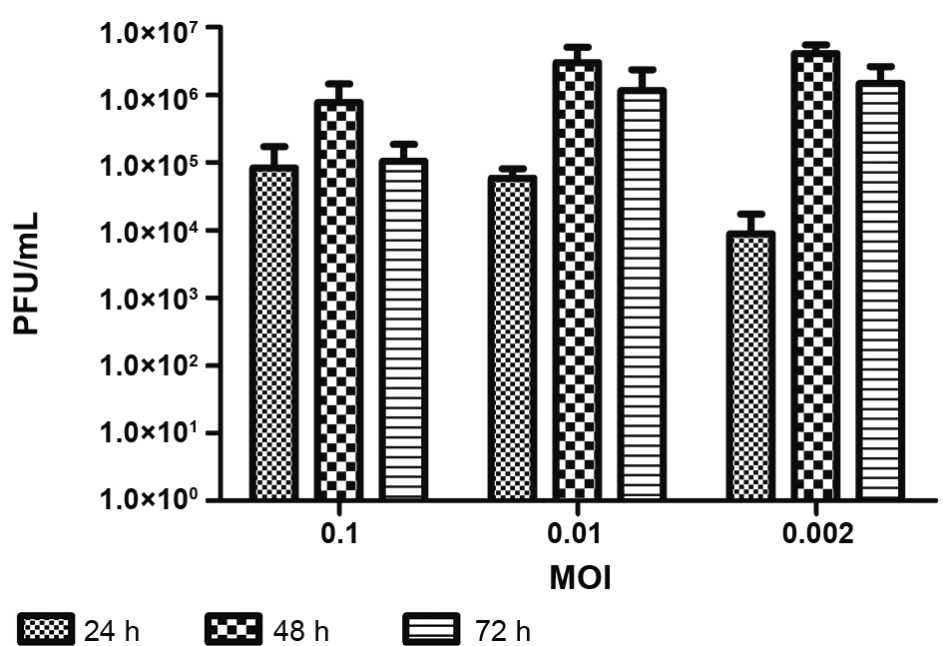
production of Yellow Fever (YF) infectious viral particles in the muscle cell cultures. Chicken embryo muscle cells infected by Yellow Fever virus (YFV) at plating stage (T0) at 0.1, 0.01, and 0.002 multiplicity of infection (MOI) were evaluated 24, 48, and 72 hpi, titrated by the plaque-forming unit assay (PFU) technique. Each bar represents a pool of three samples in triplicate.

**Fig. 8:**
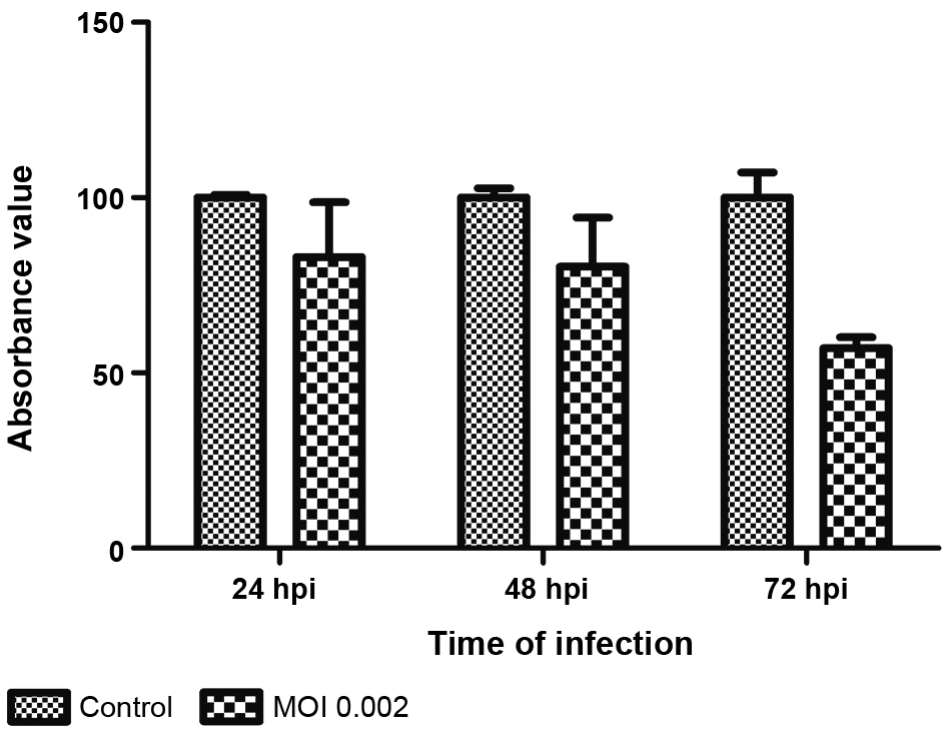
YF17DD infection leads to decrease of muscle cell density in culture. Chicken embryo muscle cells infected at the time of plating (T0) at 0.002 multiplicity of infection (MOI) were evaluated at 24, 48, and 72 h after infection by crystal violet staining. Twenty-four and 48 hpi infected cultures demonstrated loss of about 20% of cells compared to control cultures. This loss increased to 43% at 72 hpi. Absorbance reading was performed with a spectrometer at 595 nm.

*Analysis of virus production by muscle cell culture when infected with different* MOIs - To analyse if muscle cells were also susceptible and permissive to infections in lower MOIs, both 0.01 and 0.002 MOI were tested in addition to 0.1 MOI. For these analyses, only the T0 protocol was performed. Infection with 0.01 MOI was morphologically similar to 0.1 MOI at 24, 48, and 72 hpi (Fig. 6A-C), strongly contrasting to what was observed with 0.002 MOI (Fig. 6D-F). Infection with 0.002 MOI presented the most efficient production when compared to 0.1 and 0.01 MOI (Fig. 7). At 24 hpi, only a few infected myoblasts (Fig. 6D, pink arrow) were observed, but at 48 hpi, almost all muscle cells in culture were infected (Fig. 6E, green marker), followed by intense cytopathic effect at 72 hpi, when only non-infected fibroblasts survived (Fig. 6F, green arrows). These results corroborate cell densities observed in the culture during the course of infection. At 24 and 48 hpi, an approximately 20% decrease in cell density of infected cultures was observed compared to control cultures. This loss increased to 43% at 72 hpi and could be associated with cytopathic effect, demonstrated by morphological data (Fig. 8). Infection was confirmed by detection of genomic viral RNA and intermediate replicative RNA [Supplementary data (Figure)].

Corroborating morphological data, initial production of infectious particles at 24 hpi was observed. The highest level of production was observed at 48 hpi, followed by a decrease of viral particle production at 72 hpi (Fig. 7).

## DISCUSSION

In the present study, we demonstrated that chicken skeletal muscle cells are susceptible to *in vitro* infection by YF17DD virus, and that Pax7 positive cells play a central role in muscle infection both *in vivo* and *in vitro*. This model reproduces *in vivo* chicken infection, in which muscle fibres and myogenic progenitors are infected, thus corroborating previous data demonstrated by our group.^7^ ^,^ ^8^ In these previous studies, staining of viral proteins in perinuclear clusters was observed; after muscle fibre striations, a pattern was also observed here. Manso et al.^8^ discuss that the first cells to appear infected showed morphology suggestive of myoblasts. We showed by Pax7 immunostaining of tissue samples that these cells are actually myogenic precursors, indicating that they are committed to myogenic lineage but have not yet entered the myogenic program. Preferential infection of myogenic precursor cells has also been observed in Chikungunya virus.^12^ Susceptibility of Pax7^+^ myogenic precursors was also observed *in vitro*, although most cells undergo differentiation into myoblasts or myocytes in this system. However, it is possible that these cells were infected in the precursor stage, following the differentiation program. At observation time, they entered the myogenic program, failing to express Pax7 factor^13^.

Regarding the establishment of muscle cell culture, cells that are isolated by trypsinisation of skeletal striated muscle from chicken embryos have spherical morphology and quickly adhere to the substrate.^14^ In our experience, this adhesion occurs at approximately 4-5 h on a 2% gelatin coating. Adhesion is confirmed by shaking the plate and seeing that the cells seem static. Initially, these cells have an intense mitotic and migratory behaviour, and soon after adhesion, some cells begin to present an elongated shape, which will fuse to form myotubes.^14^ The time at which fusion occurs *in vitro* may vary according to medium composition and cell density.^10^ Usually, within 17 h of plating, chicken embryo myoblasts enter G0 and begin to align. At 24 h after plating, well-demarcated spindle-shaped cells can be observed, and from this point the fusion of these cells begins to form multinucleated fibres.^10^ The fusion peak occurs within a 10-h period, starting 20 h after plating, at approximately 20-30 h of culture.^15^ However, 43 h after plating, this fusion can still be observed.^10^ At 72 h after plating, cells have finished fusing and the culture is completely differentiated.^10^ ^,^ ^15^ The greatest maturation of these newly formed fibres occurs later, around one week after plating.^16^ It is possible to determine each phase of cell differentiation based on the transcription factors being expressed,^13^ cell morphology,^16^ ^,^ ^17^ and the expression of muscle-specific proteins on the cytoskeleton.^18^ Importantly, primary muscle cell culture is a mixed culture composed of fibroblasts and muscle cells. Fibroblasts can be identified by their morphology and by the absence of muscle markers.

We also determined the best harvest point of viral particle production, the best MOI, and the best infection protocol. Analysing *in vitro* infection kinetics independently of inoculation protocol or MOI used, the initial production of viral particles occurs in the first 24 hpi, and the highest production level happens at 48 hpi, followed by a titre decrease at 72 hpi. These kinetics resemble what can be observed in influenza A infection.^19^ YF viral particles produced by muscle cells hold infectious potential, able to infect new cultures. Cell death observed at 72 hpi corroborated the decrease in cell density at this point of infection, showing a direct relationship between cytopathic effect, reduction of cell density, and decrease of infectious viral particles. The most effective production of these particles was achieved by the lowest MOI inoculation (MOI 0.002). Since the inoculations with 5x (MOI 0.01) or 50x (MOI 0.1) viral particles produced no statistical difference, MOI 0.002 is the best relation between inoculum and viral production. This may be due to lower virus concentrations inducing lower levels of interferon production, which may allow a more appropriate establishment of infection. With regard to the timing of infection, even though there is no statistical difference between protocols, the T0 protocol presented slightly higher titre at 48 hpi compared with T5 and T24 at 48 hpi. The T0 protocol also seems to be a better choice for vaccine production due to less required manipulation. *In vivo* interaction between the virus and muscle cells is best represented by the T0 infection protocol, since the cells were newly isolated from the tissue. Therefore, the slightly greater viral titre in this protocol can be explained by the fact that the muscle cells are in their most naive state. As the culture proceeds, these cells tend to differentiate. At T5, by contrast, cells adhered to the culture plate and at T24, muscle cells aligned, preparing to fuse.^10^ Therefore, the slightly higher titre in T0 reinforces the hypothesis that less-differentiated muscle cells may play an important role in YFV infection.

In light of these results, we sought to understand whether undifferentiated muscle cells are necessary for the establishment of infection, or if mature muscle fibres become infected independently of immature cells. To achieve this goal, we infected muscle cell cultures after 120 h of plating (T120), when these cultures are composed of fibroblasts and muscle fibres, but myoblasts, myocytes and myogenic progenitors are no longer observed.^10^ Since infected cells were found after 48 hpi in this condition, more-differentiated muscle cells (fibres) are also susceptible to the infection. The success of infection in this protocol suggests that muscle cells are susceptible to infection at all stages of differentiation; myogenic progenitors and myoblasts do not seem to be essential for establishment of infection, although infection starts preferentially in very undifferentiated cells.

In addition to providing a better understanding of the YF17DD viral infection of muscle cells in chicken embryos, our data could be key to developing an alternative method to produce a yellow fever vaccine. Demonstration of susceptibility and permissiveness of chicken embryo skeletal muscle cells *in vitro* by YF17DD virus is described here for the first time. Some attempts based on a cell culture system have been unsuccessfully tested using chicken embryo fibroblasts (CEF) and Vero cells.^20^ ^,^ ^21^ ^,^ ^22^ ^,^ ^23^ Regarding aspects of vaccine production, the chicken muscle cell lineage is not suitable for vaccine production once immortalised with the simian vacuolating virus 40 (AcceGen Biotech cat.#ABI-TC300D). Thus, primary culture from chicken embryo muscle cells is an interesting model because it maintains the animal model used in current production, reproducing *in vitro* what occurs *in vivo*. The production of yellow fever vaccine in a cell culture system presents advantages over production in embryonated eggs since it would decrease production costs, reduce the number of animals euthanised, and lower the amount of chicken protein per dose.^20^ Altogether, it is possible that chicken embryo skeletal muscle cells can sustain the production of YFV, initially on a small scale. Nevertheless, improvements must be made to this production process to expand the capacity of viral particle production by muscle cells in culture.

*In conclusion* - The skeletal muscle cell culture model seems to reproduce the pattern of infection observed *in vivo*, suggesting that it would be a satisfactory infection model to study YFV, as well as a potential model for developing a vaccine candidate.
