# Supplementary material for: Generation of Yellow Fever virus vaccine in skeletal muscle cells of chicken embryos
Source: Mem Inst Oswaldo Cruz. 2019 Dec 9;114:e190187. doi: 10.1590/0074-02760190187 (PMC6903807; doi:10.1590/0074-02760190187)
Supplement: Supplementary file 1 [file 1678-8060-mioc-114-e190187-s.pdf]

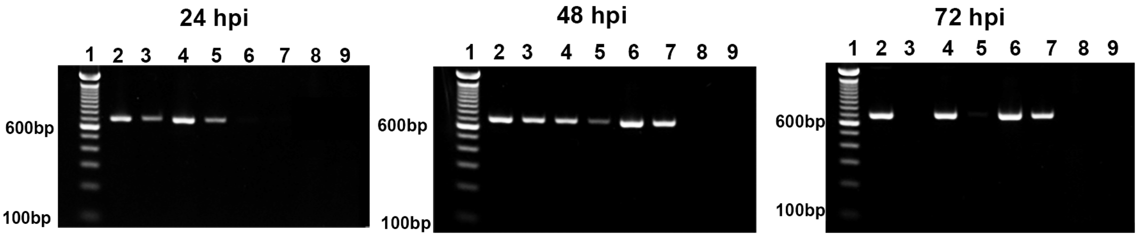

Susceptibility of culture to infection by YF17DD virus was confirmed by detection of viral genomic RNA and replicative intermediate by reverse transcription-polymerase chain reaction (RT-PCR). Detection of genomic and replicative intermediate RNA from 17DD Yellow Fever virus (YFV) extracted from skeletal muscle cells of chicken embryo infected *in vitro* at plated moment (T0) at 0.1, 0.01, and 0.002 multiplicity of infection (MOI). Polymerase chain reaction (PCR). 1: standard of 100 bp, 2: RNA genomic 0.1 MOI; 3: replicative intermediate 0.1 MOI; 4: RNA genomic 0.01 MOI; 5: replicative intermediate 0.01 MOI; 6: genomic RNA 0.002 MOI; 7: replicative intermediate 0.002 MOI; 8: Total RNA extracted from the control culture with genomic primers; 9: Total RNA extracted from the control culture with replicative intermediary primers.

BY
